# Mechanisms of Atomization from Rotary Dental Instruments and Its Mitigation

**DOI:** 10.1177/0022034520979644

**Published:** 2020-12-16

**Authors:** A. Sergis, W.G. Wade, J.E. Gallagher, A.P. Morrell, S. Patel, C.M. Dickinson, N. Nizarali, E. Whaites, J. Johnson, O. Addison, Y. Hardalupas

**Affiliations:** 1 Department of Mechanical Engineering, Imperial College London, London, UK; 2 Centre for Host-Microbiome Interactions, Faculty of Dental, Oral and Craniofacial Sciences, King’s College London, London, UK; 3 Centre for Oral, Clinical and Translational Sciences, Faculty of Dental, Oral and Craniofacial Sciences, King’s College London, London, UK; 4 Dental Directorate, Guy’s and St Thomas, NHS Foundation Trust, London, UK

**Keywords:** aerosol, SARS-CoV-2, infection control, aerosol-generating procedure, dental drill, imaging

## Abstract

Since the onset of coronavirus disease 2019, the potential risk of dental procedural generated spray emissions (including aerosols and splatters), for severe acute respiratory syndrome coronavirus 2 transmission, has challenged care providers and policy makers alike. New studies have described the production and dissemination of sprays during simulated dental procedures, but findings lack generalizability beyond their measurements setting. This study aims to describe the fundamental mechanisms associated with spray production from rotary dental instrumentation with particular focus on what are currently considered high-risk components—namely, the production of small droplets that may remain suspended in the room environment for extended periods and the dispersal of high-velocity droplets resulting in formites at distant surfaces. Procedural sprays were parametrically studied with variables including rotation speed, burr-to-tooth contact, and coolant premisting modified and visualized using high-speed imaging and broadband or monochromatic laser light–sheet illumination. Droplet velocities were estimated and probability density maps for all laser illuminated sprays generated. The impact of varying the coolant parameters on heating during instrumentation was considered. Complex structured sprays were produced by water-cooled rotary instruments, which, in the worst case of an air turbine, included droplet projection speeds in excess of 12 m/s and the formation of millions of small droplets that may remain suspended. Elimination of premisting (mixing of coolant water and air prior to burr contact) resulted in a significant reduction in small droplets, but radial atomization may still occur and is modified by burr-to-tooth contact. Spatial probability distribution mapping identified a threshold for rotation speeds for radial atomization between 80,000 and 100,000 rpm. In this operatory mode, cutting efficiency is reduced but sufficient coolant effectiveness appears to be maintained. Multiple mechanisms for atomization of fluids from rotatory instrumentation exist, but parameters can be controlled to modify key spray characteristics during the current crisis.

## Introduction

A key challenge for the return of global health care systems to “business as usual” is the inherent risk of severe acute respiratory syndrome coronavirus 2 (SARS-CoV-2) transmission via emitted sprays (including aerosols and splatter) associated with commonly performed medical and dental procedures. While barrier protection can shield health care providers, the contamination of clinical environments by sprays has led to a need to institute periods of “fallow time,” between appointments, to protect patients and staff. Fallow times are dictated by the estimated persistence of an aerosol, which is an inherently multifactorial phenomenon and restricts the use and access of a defined space. Variables include room volume, air exchange rates, airflow vectors, temperature, humidity, and the complex characteristics of the generated aerosol itself. In dentistry, the lack of robust evidence regarding the nature of procedurally generated sprays, contaminated with respiratory or oral fluids, has led to the instigation of extended fallow times, which can challenge the economic viability of current care provision models, as well as restrict patient access to care and the nature of care that can be provided.

At the onset of the coronavirus disease 2019 (COVID-19) crisis, global dental care was effectively reduced to basic management of acute needs, with a focus on exodontia when advice, analgesics, and antibiotics (3As) were insufficient to address pain (CDO-Wales 2020; Hurley et al. 2020; NHS-Scotland 2020). With a deepening understanding of the new virus, the evidence base supporting isolation, distancing, and personal protective equipment policies has been iteratively developed for societal living and health care. While much has been gleaned from the medical setting and from population-level transmission modeling, the evidence to support policies specific to the transmission risk associated with operatory dental practice is insufficient (Innes et al. 2020). Contrarily, the study of dental sprays itself is not new, and rudimentary methods to measure the spread of biological materials (blood products and culturable bacteria) during dental procedures have been reported for over 30 y. These investigations identified that many routine dental procedures, including cutting of tooth structure and dental cleaning, can spread material that is potentially infectious throughout the operatory environment (Micik et al. 1969). Since the emergence of SARS-CoV-2, new studies have replicated these findings, testing contemporary instrumentation and procedures (Allison et al. 2021). However, there has been a dependency on methods that sample dental sprays with point-based measurements using capture plates/surfaces or directional particle size counters, which have inherent limitations in generalizability across settings. Key information relevant to potential SARS-CoV-2 transmission is inadequately reported, including data on ranges of droplet size, emission trajectories, and droplet lifetimes. Importantly, identification of which procedures are intrinsically “atomizing,” producing the smallest droplets with highest latency, and those that produce high-velocity larger droplets, which may result in formites at distance surfaces, is poorly understood at the mechanistic level (Bourouiba 2020).

SARS-CoV-2 transmission in the community occurs primarily through expiratory emission of mucosalivary droplets during coughing, sneezing, or forced vocalization, such as shouting or singing (Ather et al. 2020), and can be spread by asymptomatic individuals. The virus is concentrated in saliva, with infected patients having between 9.9 × 10^2^ and 1.2 × 10^8^ viral copies/mL (To et al. 2020) and is again detectable in asymptomatic individuals (Wyllie et al. 2020). In dentistry, the use of high-velocity air and water streams is essential to cool rotary instrumentation used to cut enamel and dentin or high-frequency instrumentation used for dental cleaning. These products can combine with saliva and inevitably cause emission in the form of structured sprays with a high level of procedure-associated variability (Dutil et al. 2008; Adhikari et al. 2017; Abramovitz et al. 2020). Although the mucosalivary fluids are diluted considerably by the introduced coolants, in contrast to a respiratory emission, the operatory spray is produced over extended time periods, thus generating a potentially significant exposure.

A seminal publication at the outset of COVID-19 from Lydia Bourouiba (2020) highlighted the complexity of multiphase flows in the context of respiratory emissions. The conventional wisdom that risk assessment should be based on discrimination of large and small droplets was challenged, and it was concluded that understanding of the turbulent gas cloud dynamics must influence mitigation steps. Here we report, using high-speed imaging and quantitative flow analyses, the characteristics of dental sprays produced using high-speed rotary instrumentation and identify the mechanisms leading to atomization and ejection of high-velocity droplets. We highlight that by understanding these fundamental mechanisms, generalizable conclusions that are independent of the operatory setting may be made to inform spray mitigation. We propose that modification of operating parameters for rotary instrumentation (speed and coolant) can favor the formation of low-velocity large droplets that have low probabilities of extending beyond the immediate proximity of the patient. This, in conjunction with the prevention of mixing of the introduced spray with mucosalivary fluids using physical barriers (rubber dams) (Samaranayake et al. 1989) and capture of the spray locally with high-volume aspiration, may represent a demonstrable reduction in transmission risk.

## Methods

Sprays generated from conventional air turbines (W&H; with water coolant and “chip air,” ~450,000 rpm) and an electric micromotor (NSK; with water coolant with and without “chip air”) with 5:1 speed increasing hand-sets (X95L; NSK) were parametrically studied (from 20,000 to 200,000 rpm at 20,000-rpm intervals). Measurements were undertaken in an unobstructed steady-state mode, with burr contact to dental enamel (1.5 ± 0.5 N), and in intraoral simulations using a modified dental training mannequin (buccal spaces blocked out with absorbent wadding and floor of mouth with a polyvinylsiloxane “tongue” to result in a cavity volume of ~100 mL) with all surfaces prewetted with water before measurements to simulate oral fluids. The measurements took place within a maximum distance of ~0.2 m from the generating spray source. This ensured a satisfactory signal-to-noise ratio in the imaging process and increased the overall capturing capacity of the emitted spray that would have been otherwise reduced due to the limitations imposed by its high directionality and airflow conditions, especially at a distance from the source.

A coherent and directional continuous-wave 450-nm laser beam, a multidirectional 450-nm LED light source, and broadband wavelength light were used to illuminate the sprays. For the laser measurement cases, the illumination was achieved through laser sheet–forming optics that provided a “2-dimensional” illumination plane along the axial propagation axis of the sprays. The sprays were captured with the use of a high-speed camera (Photron FASTCAM Mini AX200 type 900 KM 32 GB) at variable angles, distances, and frame rates. All broadband light and LED-illuminated sprays were recorded with frame rates ranging between 1 and 2 kHz through Photron’s Fastcam Viewer (PFV) software. PFV was also used to control the camera for these measurements. All laser-illuminated sprays were recorded with a frame rate of 0.5 kHz through LaVision’s DaVis software. DaVis and a LaVision PTU-X external unit were used to trigger and control the camera. The minimum spatial resolution achieved with the current setup is 100 µm. It must be noted that even though the spatial resolution of our instruments could not detect individual droplets less than about 100 µm, collectively, these clouds of smaller droplets appear in the recording as mist, which can be quantified.

Preliminary estimates of the presented droplet velocities were calculated via PFV from the broadband and LED illumination cases. The probability density maps for all laser-illuminated spray cases have been processed using DaVis. First, 2,000 raw images for each test case were dewarped via the use of a calibration plate. The images were subsequently binarized based on a trial-and-error intensity thresholding process. The intensities from all spray locations (pixels) for every image recorded in a given case study were counted and divided by the number of images used for every test case. This allowed the generation of spatial probabilistic distributions of the spray for every test case measured. MATLAB (R2020a; MathWorks) was subsequently used to quantify the probability distributions of the spray for each half of the view field upstream and downstream of the spray release locations. The laser sheet–illuminated sprays and associated calculations systematically underestimate the spatial quantification of the spray due to the presence of a shadow cone cast by the burr tip, fluid film, and droplets (blanked white space underneath burr at the bottom-right corner of the imaging plane). Nevertheless, a systematic and parametric comparison between the cases is possible.

An optical particle size counter (Model 330; TSI Incorporated), with a range from 0.3 to 10 µm and size resolution of <5% at 0.5 µm, was used to supplement optical measurements. The instrument was placed 1.5 m from the emission source. Following baseline measurements, handpieces were fixed with burr-to-tooth contact and run continuously for 1 min. After a further 30 s to allow dispersal, data were collected (taking a further 1 min). In all cases, baseline conditions were reestablished before further measurements were performed with typically 3 independent repeats per condition. The impact of varying the coolant parameters on heating during instrumentation was considered, and methods and results are presented in the Appendix.

## Results

**Figure 1.**
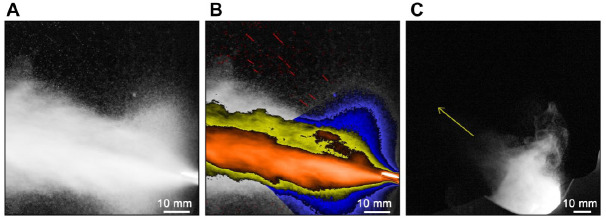
Air turbine visualizations using broadband and LED light sources. (**A**) Still frame from high-speed imaging of an unobstructed spray from an air turbine possessing a high-velocity spray, a turbulent shear layer at the periphery of the core, and recirculation regions close to the burr tip. (**B**) Same image false-colored with the central spray core in orange, a shear layer region located outside of the central core region in yellow, and recirculation regions in blue. A group of high-droplet velocity straight trajectories is shown by the red streaks toward the top of the image. (**C**) When the same instrument is placed inside a simulated oral cavity (palatal to the maxillary central incisors), a turbulent fine mist of a reduced but significant velocity is produced (principal direction indicated by the yellow arrow).

Dental rotary instruments facilitate the use of high-speed rotating burrs that can apply concentrated frictional forces to remove tooth structure quickly. The devices have been traditionally powered by low-torque micro–air turbines (Pelton wheels) and more recently by electric micromotors, with the former requiring high rotational speeds of ~0.5 million rpm to counteract their lack of torque. Water is used to remove ablated debris, cool internal moving parts, and prevent overheating of the dental pulp. In general, for both devices, air is premixed (so called “chip air”) with water in the instrument head to “premist” the coolant, creating a high-velocity flow that exits the handset from multiple holes in its base. It is then directed to the burr tip and into the mouth in the form of a dense and fine spray. The high-speed, fine-mist spray is subsequently modified by its interaction with the rapidly rotating burr tip. When this spray is unobstructed (Fig. 1A), it is seen to project at speeds that can exceed 12 m/s. Millions of small droplets are generated, which, because of their small mass, have limited gravitational effect on their trajectories. For comparison, peak exhalation velocities from sneezing have been quantified in a range 10 to 30 m/s. In Figure 1A, the spray from the air turbine can be seen to increase in a cross-sectional area as it disperses through the air at a shallow angle from the generation origin. A fast-moving fine-mist spray core is formed, surrounded by a shear layer region, where the spray interacts with the surrounding air. The high-speed spray causes entrainment of the surrounding air and a recirculation vortex extending at the base of the handset (top and bottom from the origin of the spray). This region is formed in its majority by a fine mist and is indicated by the blue region of Figure 1B. Larger droplets, with high velocities, are randomly ejected due to the physics of the atomization process (appearing as red streaks in Fig. 1B). These droplets move along straight trajectories due to their inertia being unaffected by the airflow and therefore are not contained in the main body or recirculation regions of the spray. Such droplets are unlikely to be consistently detected using point-measurement approaches due to the stochastic nature of their formation but carry a significant mass of the introduced water flowrate.

A key problem in modeling dental procedurally generated sprays is the enormous heterogeneity in outcome due to the positioning of the generation source by the clinician as they operate on different aspects of teeth across the oral cavity, together with the interaction of the introduced flow with the mouth, its structures, and its pooled mucosalivary fluids. Figure 1C emphasizes this, showing that the spray morphology is immediately perturbed when directed into the oral cavity (albeit here in a controlled training mannequin with simulated oral fluids and aspiration). In general, obstruction of the direct flow reduces droplet velocities and extent of spread, but a significant and notable fine mist of droplets persists with velocity, density, and direction all dependent on the positioning of the tool.

**Figure 2.**
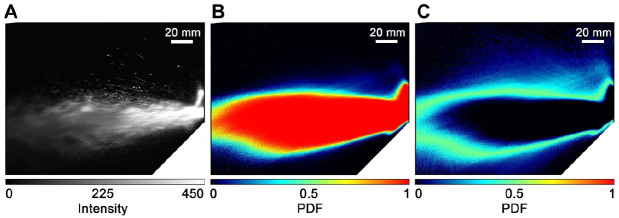
Air turbine visualizations using laser sheet optics. (**A**) An instantaneous image of the spray formed by an air turbine unobstructed running in a steady state. (**B**) Probability distribution of the spray droplet concentration based on >2,000 images. Pixels that are red indicate a 100% chance of encountering a droplet at any point in time, and pixels that are black represent 0%. (**C**) Standard deviation, plotted on an equivalent scale.

Figure 2 illustrates the quantitative method used to study spray generation in this study showing a single image frame in Figure 2A, the probability density map from >2,000 images in Figure 2B, and its associated standard deviation in Figure 2C for an unobstructed spray generated by an air turbine. A dense spray occupying the majority of the image field is produced with droplet velocities that can exceed 12m/s. Data are lost from a region under the burr tip due to a shadowing of the illuminating light sheet, and this represents an underestimation in the near-field characterization of the spray. This is consistent throughout all measurements reported here.

**Figure 3.**
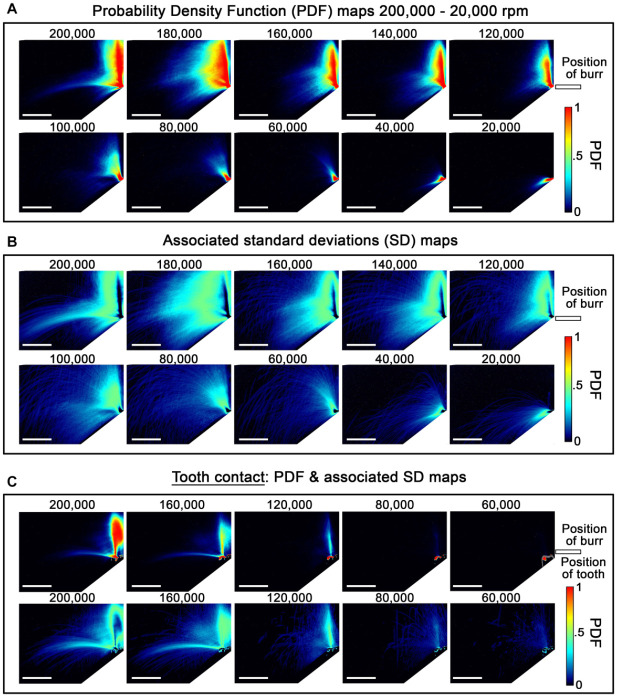
Quantification of spray generation using laser sheet optics. (**A**) Probability density function (PDF) and (**B**) associated SD maps of droplet concentration for a micromotor with a 5:1 speed-increasing handpiece run with no “chip air” rotating between 200,000 and 20,000 rpm. PDF maps are based on >2,000 images for each modality with the instrument running unobstructed in steady state. At 200,000 rpm, most of the droplet velocity is ~1.4 m/s, which is an order of magnitude less than an air turbine. PDFs of droplet concentration (**C**, top line) and SD (**C**, bottom line) of the concentration fluctuations for a micromotor run with no “chip air” rotating at decreasing speed with the burr tip in contact with wet enamel. Distributions are again based on >2,000 images for each modality. Spray distribution and trajectories are modified by tooth contact. There is evidence at higher speeds that atomization near the tooth surface occurs; however, with decreasing speed, the coolant largely streams over the tooth surface with a limited number of low-velocity droplets being deposited within the imaging field of view. Scale bars are equivalent to 50 mm in all images.

**Figure 4.**
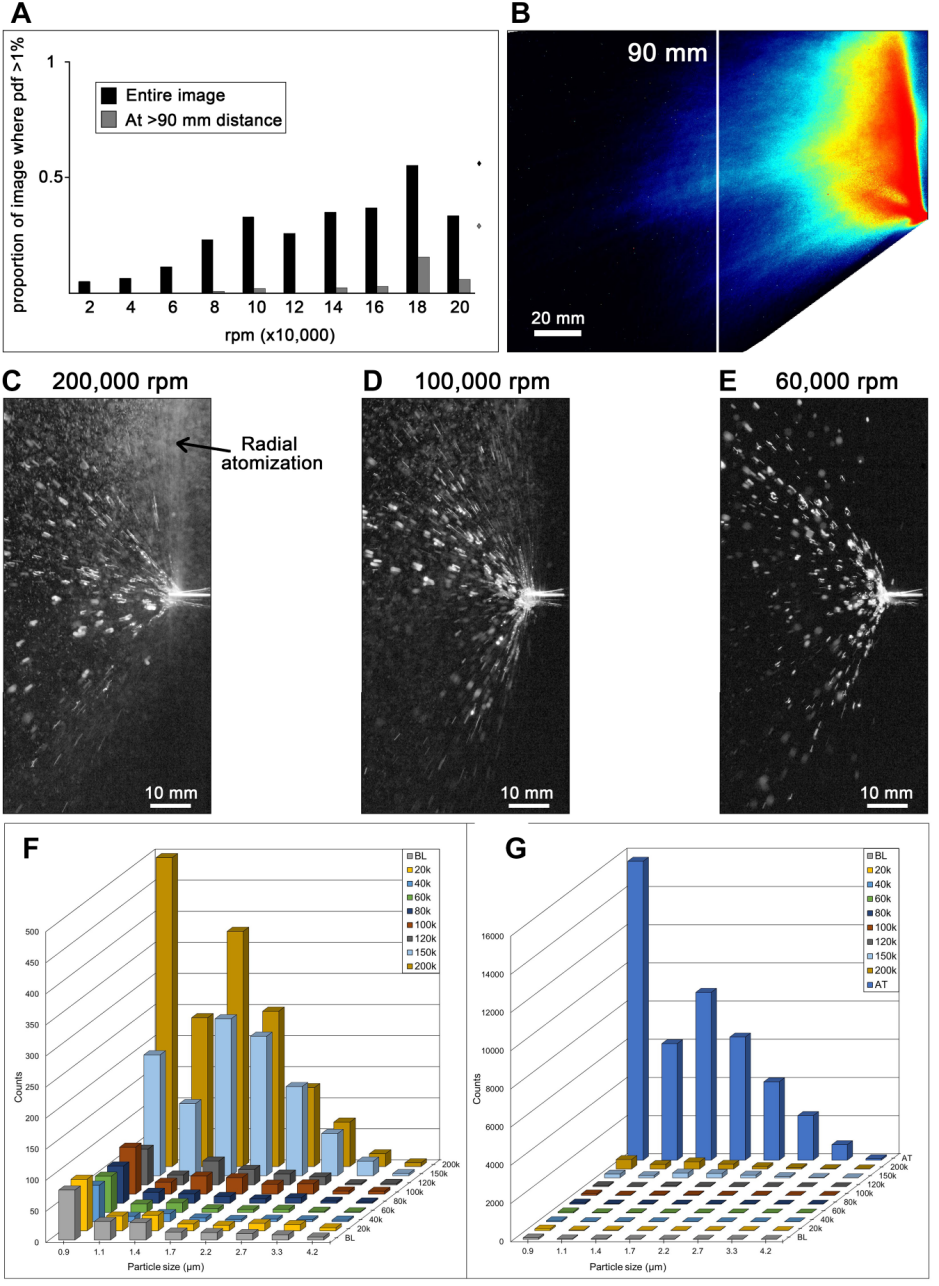
Quantification of spray generation as a function of speed of burr revolution. (**A**) Proportion of image field where the probability density function of droplet concentration is greater than 1% plotted against micromotor speed, for the entire imaging field in (**B**) (black-colored bars) and the left side of the imaging field (gray-colored bars), which is ~90 mm from the burr tip. For comparison, equivalent values for an air turbine are plotted as diamonds at the right of the histogram. We observe that reduction of micromotor speed to 60,000 rpm results in, at a distance of >90 mm away from the burr, only 0.1% of the imaged pixels having >1% chance of encountering a droplet. This represents a ~280-fold reduction compared with an air turbine. (**C–E**) Representative sprays with an elimination of radial atomization below 100,00 rpm. (**F**, **G**) Histograms showing droplet particles sizes associated with non-premisted micromotor speed (rpm) compared with ambient baseline (BL) and air turbine (AT) with premisted coolant.

**Figure 5.**
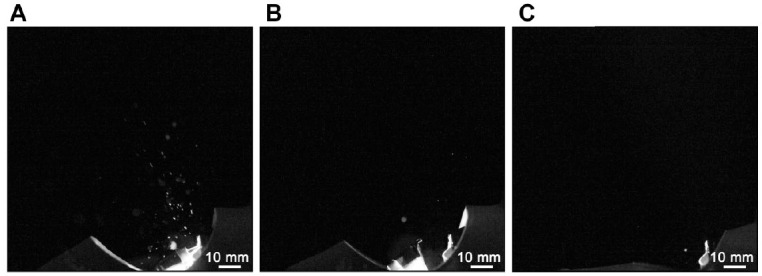
Representative images showing spray formation when mechanisms identified to cause atomization are mitigated, achieved here by running an electric micromotor with a 5:1 speed-increasing handpiece with “chip air” blocked and revolutions restricted to 60,000 rpm. The burr was held in contact with the palatal surface of right maxillary central incisor (**A**), lingual surface of right mandibular central incisor (**B**), and the occlusal surface of the right mandibular first molar (**C**).

When air and water are “premisted” prior to delivery of the coolant to the burr tip (for both air turbines and electric motor handsets using “chip air”), atomization occurs due to the shear introduced by the high-pressure air splitting droplets apart. To consider whether forces introduced by the rapidly rotating burr tip can also atomize, the “chip air” was blocked for the electric motor with the speed-increasing handset and the spray studied as a function of decreasing revolution speed. In Figure 3, it can be seen that the spread of the spray significantly reduces with decreasing rpm, but in the plots of the standard deviation of the probability density function, it can be seen that a combination of both atomized spray (fine droplet) and higher-velocity droplets is produced at a speed above 100,000 rpm. This confirms that the fluid interaction with the rotating burr tip can result in radial atomization, but a threshold (for the system studied) exists between ~80,000 and 100,000 rpm, in which a reduction in droplet velocity and an increase in droplet size (reflected as an increase in droplet trajectory curvature) were observed. Spray distribution and trajectories are further modified by tooth contact, and in Figure 3, it can be seen that atomization occured at the tooth surface at revolutions >80,000 rpm, likely due to the impact of liquid fragments on variable-thickness liquid films formed at the tooth surface, which has significant implications for atomization of thin salivary films also present on tooth surfaces. Trends observed in Figure 3 are quantified in Figure 4A, B and can be directly visualized in single-frame images in Figure 4C–E, which shows the generated spray at 60,000 rpm comprising only larger, slow-moving droplets all appearing to follow parabolic trajectories (under gravitational influence) within the imaging field. In Figure 5, it is seen that when the mechanisms understood to result in atomization are mitigated, achieved here by running an electric micromotor with a 5:1 speed-increasing handpiece with “chip air” blocked and revolutions restricted to 60,000 rpm, in all clinically simulated positions, the spray generated is minimal, with no evidence of misting. Optical particle size measurements summarized in Figure 4F, G and in Appendix Table 1 show good agreement with optical data, with droplet particle sizes <5 µm only detectable above baseline levels at revolutions >80,000 rpm. Introduction of premisting of the coolant for the air turbine led to a dramatic increase in all detectable particle sizes.

## Discussion

The complexity, particularly the heterogeneity of generated dental sprays (including aerosols and splatter), underpins the great challenges faced by the dental profession and the risk to individuals, while community levels of risk for SARS-CoV-2 transmission remain at a tangible level. The so-called universal precautions used in dental operatory settings to protect patients and care providers were introduced primarily in response to the emergence of blood-borne viruses, including HIV. Unfortunately, as with so many areas of health care, dentistry has not prepared for the possibility of a viral respiratory pathogen that has potentially high transmittance within a dental care setting. Academic systematic review has highlighted the paucity of high-quality evidence (Innes et al. 2020). Accordingly, there is a paucity of robust evidence to support policy makers. Notably, a recent Cochrane review, published to address concerns related to risk of SARS-CoV-2 transmission in dental settings, has failed to identify adequate scientific evidence, leaving dental regulators to base policies on “expert” opinion, with interpretation of the same evidence by different bodies often being contradictory (Verbeek et al. 2020).

A significant point of contention has been how to define dental sprays and extrapolate risk based on the emerging understanding of SARS-CoV-2 transmission. Historically in dentistry, aerosols have referred to droplets less than 50 µm in diameter, of which those <10 µm were considered an inhalation risk (Micik et al. 1969). This is a disparity with accepted respiratory infection classifications, and for SARS-CoV-2 transmission, virally loaded airborne droplets <5 µm that remain suspended in the air for prolonged periods are considered of particular risk (Cook 2020) alongside “splatter” of infected larger droplets that have the potential to contaminate fixed and mobile surfaces and are subsequently introduced into respiratory or ocular systems due to inadequate hand hygiene (Szymanska 2007). In this study, we demonstrate the underpinning mechanisms that lead to the formation of these features of dental sprays formed by rotary instrumentation.

We have identified 4 contributing mechanisms to the atomization of coolant water and/or a mixture of coolant water and oral fluids by rotary handsets in the form of multiscale droplet sizes (including mist) and velocities. These are as follows:

1. Premisted and premixed cooling water and air generated internally by air turbines and micromotors operated with chip air. A high-velocity mixture of air and droplets is ejected through ports at the base of handset heads and directed to the burr tip of the instruments for cooling purposes during tooth ablating/polishing.
2. For the unobstructed cases (i.e., when the burr tip is not interacting with a tooth surface), interaction of the high angular velocity burr tips with the
  - sprayed coolant water film established on the burr for cooling purposes,
  - other droplets, and
  - pooled oral liquids
  leads to droplet formation and ejection of high-speed “projectile” droplets radially or at a forward angle to the burr rotational axis and coolant ejection direction.
3. Interaction of the high angular velocity burr tips with the
  - sprayed coolant water film established on the burr for cooling purposes,
  - other droplets, and
  - pooled oral liquids
  and the tooth surface leads to an increased induced liquid shear layer between the rotating burr tip and the tooth, causing fine misting of liquids within the layer and high-speed “projectile” droplets. The droplets and mist are ejected stochastically according to the orientation of the interacting surfaces.
4. Secondary processes that might cause liquid atomization because of high-speed primary atomized droplet and/or air/droplet mixture collision/interaction with other droplets, liquid films, or pooled liquids within the mouth cavity are expected to generate low-speed, large-size droplets.

The generalization of findings of dental spray generation studies into the wide variety of clinical settings that exist is complex, particularly when the spray itself can be so variable. The speed, direction, size, and number of droplets emerging from the oral cavity for each handset are different and expected to change according to the type, location, orientation, and specific operation of the dental instrument with respect to the interaction of the instrument and generated spray with hard and soft tissues of the oral cavity. Mixing of the introduced coolant with real saliva also requires consideration. Saliva is rheologically complex, differing according to stimulation method, physiological conditions, time, and between individuals. It is described as a non-Newtonian, shear thinning liquid with viscoelastic/pseudo-viscoelastic properties (Łysik et al. 2019) exhibiting Newtonian behavior at high shear rates (Foglio-Bonda et al. 2014). Its dynamic viscosity ranges between 1 and 20 mPas. It is expected that large dilution of saliva with excess cooling water (dynamic viscosity of 1 mPas) results in a mixture expected to be rheologically more like water and was therefore not simulated in this initial study.

Here an overarching approach to assist risk assessment with dental spray–generating procedures is reported. We observe that rotary instrumentation with high-torque electric micromotors and 5:1 speed-increasing handsets can be used without atomization or the ejection of high-velocity droplets when specific operating parameters are selected. Although cutting efficiency is significantly reduced, the machining of enamel, dentine, and some restorative materials is achievable with adequate cooling to prevent pulp injury still provided in the absence of “chip air” (Appendix Table 2, Appendix Figs. 1 and 2) when operated at reduced speeds (80,000 to 100,000 rpm). The impact of these machining protocols on thermal damage at the site of the cut substrate requires further investigation but is beyond the scope of the current study. These measures in the short term may allow many routine operatory procedures to be performed and are feasible without major infrastructural modification to surgery environments. Inevitably, risk assessments at local levels, put in context of population infection rates, mitigation factors (on which there is emerging evidence) such as barrier dams to prevent oral fluid mixing, aspiration, and air filtration and ventilation schemes, all must guide decision making, but in certain settings, such as open plan clinics that are commonly found in dental education settings, modification of instrumentation protocols is likely to be essential in the short term.

## Author Contributions

A. Sergis, O. Addison, Y. Hardalupas, contributed to conception, design, data acquisition, analysis, and interpretation, drafted the manuscript; W.G. Wade, J.E. Gallagher, S. Patel, C.M. Dickinson, N. Nizarali, E. Whaites, J. Johnson, contributed to conception and data interpretation, critically revised the manuscript; A.P. Morrell, contributed to conception, data analysis, and interpretation, critically revised the manuscript. All authors gave final approval and agree to be accountable for all aspects of the work.

## Supplemental Material

sj-pdf-1-jdr-10.1177_0022034520979644 – Supplemental material for Mechanisms of Atomization from Rotary Dental Instruments and Its Mitigation Click here for additional data file. Supplemental material, sj-pdf-1-jdr-10.1177_0022034520979644 for Mechanisms of Atomization from Rotary Dental Instruments and Its Mitigation by A. Sergis, W.G. Wade, J.E. Gallagher, A.P. Morrell, S. Patel, C.M. Dickinson, N. Nizarali, E. Whaites, J. Johnson, O. Addison and Y. Hardalupas in Journal of Dental Research
