# Supplementary material for: Mechanisms of Atomization from Rotary Dental Instruments and Its Mitigation
Source: J Dent Res. 2020 Dec 16;100(3):261–7. doi: 10.1177/0022034520979644 (PMC7746949; doi:10.1177/0022034520979644)
Supplement: sj-pdf-1-jdr-10.1177_0022034520979644 – Supplemental material for Mechanisms of Atomization from Rotary Dental Instruments and Its Mitigation [file sj-pdf-1-jdr-10.1177_0022034520979644.pdf]

“Mechanisms of atomization from rotary dental instruments and its mitigation”

Supplemental Material

Methods:

The effectiveness of cooling achieved when a micromotor with a 5:1 speed increasing handpiece was used with ‘chip-air’ blocked was assessed on a thermally conductive substrate (brass) and on bovine dentine. Temperature measurements were made using a standardized experimental set-up initially at room temperature. A 2 mm of brass plate was machined using a 541 pattern burr for each condition and K-type thermocouples placed in intimate contact with the contra-lateral surface with simultaneous monitoring of the incoming coolant temperature at 10 Hertz using a Pico TC-08 datalogger accurate to 0.1°C (note that with increasing motor use, the unit heats the incoming coolant slightly with time).

A further series of measurements were conducted with the substrate thermally held at initially at ~36.5°C to identify whether the incoming coolant (fed at a lower temperature of ~23°C) still acted to reduce overall temperature effectively. Measurements were performed on both brass and bovine dentine. Machining was stopped when ~1mm of material over the thermocouple had been removed.

Results:

Supplemental Table 1.

Size (µm)	**BL**	**20,000 rpm**	**40,000 rpm**	**60,000 rpm**	**80,000 RPM**	**100,000 rpm**	**120,000 rpm**	**150,000 rpm**	**200,000 rpm**	**Air Turbine**
0.3	4071 ± 204	3920	3937 – 3964	3388 – 3618	3210 – 3381	3158 - 4081	2960 - 4121	3127 - 5630	3556 – 5723	76118 –113734
0.38	1255 ± 86	1229	1195 - 1212	1135 - 1152	1077 - 1109	1055 - 1333	945 - 1329	943 - 1694	1212 - 1771	49408 – 65818
0.47	370 ± 87	494	259 – 489	396 – 455	335 -349	325 - 368	313 - 315	406 – 455	523-564	34360 – 48935
0.58	160 ± 7	186	152 – 191	144 – 157	126 -133	127 - 152	116 – 149	156 - 358	384-447	21383 - 36458
0.72	57 ± 14	51	39 – 72	49 -58	48 -63	47 – 58	57 – 62	82 – 169	275 - 306	11502 – 18693
0.90	80 ± 13	83	53 – 64	54 - 65	56 -67	72 – 79	52 – 63	122 – 267	458 - 536	15659 – 29598
1.12	30 ± 4	24	8 – 9	12 -15	13 - 20	18 – 20	16 – 27	69 – 163	204 - 275	6106 – 10570
1.39	28 ± 7	25	3- 23	14 -18	12 -17	28 – 30	27 – 50	164 – 341	306 - 451	8793 – 14317
1.73	13 ± 3	11	3 – 9	5 -7	8 -14	24 – 29	16 – 34	122 – 327	189 - 311	6452 – 11299
2.16	12 ± 3	9	2 – 5	2 - 7	5 -8	13 – 19	11 – 24	72 – 215	105 - 149	4107 – 7579
2.69	10 ± 3	12	1 - 7	4 - 6	6 -13	15 – 18	8.0 – 18	18 -118	56 - 87	2340 – 4897
3.34	8 ± 3	10	1 - 5	1 - 3	1 -5	4 – 6	1 – 3	5 -42	8 - 33	815 – 2682
4.16	5 ± 2	5	0 - 5	0 -1	0 - 2	3 – 6	2 - 3	2 -5	1 –11	81 – 925

Binned counts of droplet particle sizes recorded at a fixed distance of 1.5 meters from the emission source after 1 min of instrument operation for electric micromotors and speed increasing handpieces with no premisting running at 20,000 to 200,000 rpm and for an air turbine with premisting running at ~450,000 rpm. Mean and standard deviations are provided for baseline (BL) where n=6. A single measurement was obtained for 20,000 rpm and for all other values ranges are provided (n=3). Data is reproduced in Figure 4.

Supplemental Table 2.

Revolutions per minute (rpm)	200k	160k	120k	80k	60k	40k
Temp rise above coolant temp after 15s (°C)	1.5±1.1	0.7±0.4	0.4±0.1	0.5±0.4	0.4±0.1	0.4
Temp rise above coolant temp after 45s (°C)					0.9	

Coolant at 22.5-23.0 °C

Mean temperature rise (and associated standard deviation) above incoming coolant temperature following machining of standardised brass plates (2 mm thickness) with a 541 pattern burr, at various motor speeds (rpm) with ‘chip-air’ blocked preventing pre-misting of the coolant (n=3). Data demonstrates at non-atomising rotary speeds relevant to the primary objective of this report only a minor temperature rise (<1°C) was measured on the contralateral surface of the machined substrate after short and more prolonged cutting periods.

Supplemental Figure 1.

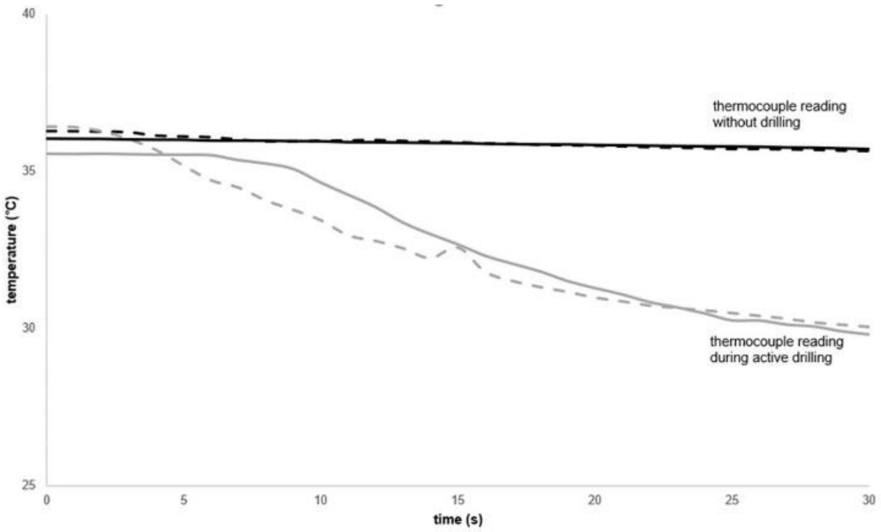

Thermal data of the test substrate held at ~35.5°C to approximate physiological temperature with and without machining at 60,000 rpm with a 541-pattern burr with ‘chip-air’ blocked. With no drilling the temperature control remained constant. Drilling for 30s with no pre-misting of the incoming water resulted in a cooling of the system plateauing as the temperature approached that of the coolant demonstrating continued effectiveness of the coolant in this experimental set-up.

Supplemental Figure 2.

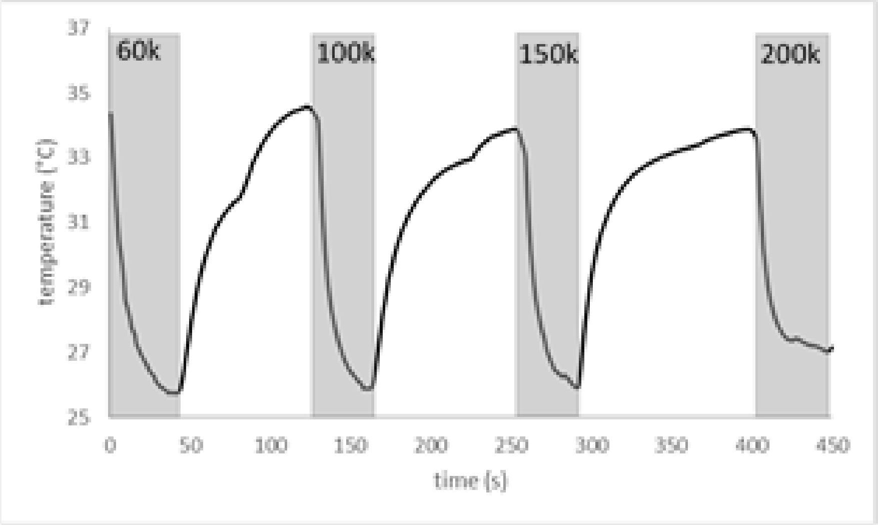

The figure shows temperature measurements made during cutting of 2 mm thickness bovine dentine plates held at a temperature of ~35°C. Regions highlighted in grey indicate active cutting of the substrate to a 1 mm minimum thickness from the thermocouple at various rpm with a 541-pattern burr with 'chip-air' blocked. Cooling effectiveness reduces with increasing rpm (60k, 100k, 150k and 200k) but a significant net reduction in the overall temperature of the substrate for each mode indicates suitable cooling is still achieved (i.e. no net increase above physiological temperature at the thermocouple position simulating the pulp).
